# A one-year prospective study on the occurrence of traumatic spinal cord injury and clinical complications during hospitalisation in North-East Tanzania

**DOI:** 10.4314/ahs.v21i2.39

**Published:** 2021-06

**Authors:** Haleluya I Moshi, Gunnevi G Sundelin, Klas G Sahlen, Ann VM Sörlin

**Affiliations:** 1 Department of Community Medicine and Rehabilitation, Physiotherapy, Umeå University, Umeå, Sweden; 2 Physiotherapy Department, Faculty of Rehabilitation Medicine, Kilimanjaro Christian Medical University College, Moshi, Tanzania; 3 Department of Public Health and Clinical Medicine, Epidemiology and Global Health, Umeå University, Umeå, Sweden

**Keywords:** Rehabilitation, pressure ulcer, spasm, urinary tract infections, low income countries

## Abstract

**Background:**

Clinical complications following spinal cord injury are a big concern as they account for increased cost of rehabilitation, poor outcomes and mortality.

**Objective:**

To describe the occurrence of traumatic spinal cord injury and associated clinical complications during hospitalisation in North-East Tanzania.

**Method:**

Prospective data were collected from all persons with traumatic spinal cord injury from North-East Tanzania from their admission to discharge from the hospital. Neurological progress and complications were assessed routinely. Data were captured using a form that incorporated the components of the core data set of the International Spinal Cord Society and were analysed descriptively.

**Results:**

A total of 87 persons with traumatic spinal cord injury were admitted at the hospital with a mean age of 40.2 ± 15.8 years. There were 69 (79.3%) males, and 58 (66.6%) of the injuries resulted from falls. Spasms (41 patients, 47.1%), neuropathic pain (40 patients, 46%), and constipation (35 patients, 40.2%) were the most commonly reported complications. The annual incidence rate in the Kilimanjaro region was at least 38 cases per million.

**Conclusion:**

The incidence of traumatic spinal cord injury in the Kilimanjaro region is relatively high. In-hospital complications are prevalent and are worth addressing for successful rehabilitation.

## Introduction

Trauma to the spinal cord may lead to death at the scene of the accident, during the acute phase, or later due to organ failure and/or clinical complications. For the past five years, systematic reviews have shown that the annual global incidence of traumatic spinal cord injury (TSCI) ranges from 3.6 to 246.0 new cases per million population 1,2. However, there is a scarcity of epidemiological studies on TSCI in low-income countries, particularly in Africa. In the sub-Saharan region, two prospective studies in South Africa and Botswana reported annual incidences of 75.6 and 13 per million population, respectively 3, 4. Although the data were inconclusive, a review of the few available reports from low-income countries suggested that the incidence of TSCI was more than 25 cases per million population annually^5^.

As soon as TSCI has occurred, there are few days or weeks of immediate risk of death due to acute autonomic dysfunction and cardiorespiratory failure 6. With comprehensive life support and rehabilitation services, this risk of acute death decreases, and the patient stabilises physiologically and psychologically with time. However, depending on the level and severity of the injury, this acute and most critical period may be followed by persistent troubling symptoms such as neuropathic pain and spasms that affect rehabilitation and quality of life in general. Neuropathic pain and spasms are among the signs and symptoms of SCI and may persist for the rest of the person's life 7, 8. Common secondary complications that are not among the signs and symptoms of TSCI include pressure ulcers (PUs), respiratory and urinary tract infections (UTIs), and deep venous thrombosis 9. It has been shown that these secondary complications elevate the risk of death in persons with SCI even after discharge from the hospital.

PUs is among the leading and most complex fatal complications in the SCI population 10. In one hospital-based prospective study in South Africa, PUs is registered as the most prevalent complications following SCI 11. Management of PUs is complex, and if the wound becomes infected death due to septicaemia may occur 12. Following a PU on the immobile, insensitive, and vascular diminished tissue, healing becomes difficult and susceptibility to infections increases. Septicaemia, which is one of the most common causes of death in persons with SCI results not only from unsuccessfully treated PUs, but also from UTIs or pneumonia^12^, ^13^.

Another route for infections is the urinary tract due to repeated or prolonged use of catheters 14. Both indwelling and intermittent catheterisation can introduce different types of bacteria to the urinary system, leading to UTIs. A recent systematic review has shown that UTIs are the leading complications among persons with SCI and are acknowledged as difficult to prevent 12. However, UTIs are not as potentially fatal as PUs. Depending on the type and extent of infection, untreated UTIs might cause irreversible destruction to the urinary system^15^. It is not uncommon for UTIs to ascend all the way to the kidneys, leading to nephritis and dysfunction of the vascular and urinary system.

Pulmonary complications are also common and account for a significant proportion of the mortality during or after the acute phase, especially in cases with high-level injuries and in elderly persons with TSCI 16. Apart from respiratory failure, which is most common in the early and most acute phase, respiratory infections such as pneumonia can be either acute or later complications. Common factors that are known to contribute to respiratory complications include high spinal level, complete injury, history of smoking, and steroid use 17. All of these TSCI-related complications are of medical and rehabilitation concern because they are associated poor functional outcomes, high rehabilitation cost, prolonged hospital stay, anxiety, depression, poor quality of life (QoL), and death 18. However, most of these complications can be prevented with adequate knowledge, skills, and equipment. Lack of the aforementioned pre-requisites accounts for the reported high prevalence of complications in low-income countries. There are very few published studies in Tanzania with regards to TSCI-related complications, thus it is difficult to inform prevention, care, and rehabilitation services. This study aimed to describe the occurrence of TSCI and prevailing complications during hospitalisation in one rural area of Tanzania.

## Methods

### Study Design

This was a descriptive one-year prospective study in which data were collected from TSCI patients on admission, during hospitalisation, and at discharge.

### Study population and setting

This study targeted all newly injured persons from north-east Tanzania admitted to Kilimanjaro Christian Medical Centre (KCMC), which is the only consultant hospital with a spinal unit in the region.

Tool for data collection

A data collection sheet based on the international SCI core data set 19, 20 was used in this study. Other variables such as associated injuries, in-hospital complications, skeletal injury, death, and ambulatory state at discharge were also added to the data collection sheet. Additionally, a chart was developed to register the anatomical location and date of starting and healing of PUs^21^. The American Spinal Injury Association Impairment Scale^22^,^23^ was used to classify the completeness of the injury.

### Procedure for data collection

All new cases of TSCI were identified from the hospital admission records within the first 24 hours of admission. TSCI was confirmed by evidence of persisting sensory and/or motor loss, which might include bladder and bowel dysfunction beyond the normal spinal shock period. The injured person was followed up in the ward, and neurological assessment was conducted as a matter of routine. All patients consented for their medical data to be used for this study. The data collection sheet included sociodemographic information, cause of injury, and the address of each patient. Each patient was inspected for associated injuries and whether or not there were skin changes suggestive of PUs. PUs was defined as any skin change due to sustained pressure that required any form of management, including a planned pressure relief procedure. Patients were asked to report any new complaint such as constipation, pain, or spasms. Each patient was followed up from admission to discharge or death.

### Data analysis

Data were cleaned and entered into the Statistical Package for Social Science (SPSS version 25) for analysis. Age, length of hospital stay (LOS), and time for PU appearance and healing were analysed descriptively, and the results are given as means and standard deviations. Pain and spasms were subjectively reported and described by the patient. Incidence based on the Kilimanjaro region population (N = 1,910,555) was calculated after subtracting children 10 years of age and younger (N = 464,600) because none of these were admitted with TSCI in 2017.

### Ethical considerations

This study was a part of a larger project on SCI in the north-east Tanzania that had ethical approval from the research ethics review committee of the KCMC, registration number 620. Patients were requested to consent to the use of their medical information for research purposes.

## Results

### Socio-demographic characteristics

For the year 2017, a total of 87 persons with TSCI were admitted to KCMC. Of these, 56 (64.4%) were from the Kilimanjaro region and 31 (35.6%) were from neighbouring regions. There were 69 (79.3%) males, and the mean age was 40.22 ± 15.77 years. Most injuries involved the cervical level (49 patients (56.3%)) followed by lumbar (27 patients (31%)) and thoracic (11 patients (12.6%)) levels. In terms of the severity of the TSCI, 35 (40.20%) patients had complete (AIS “A”) and 44 (50.6%) patients had incomplete (AIS “B, C, D”) injuries, while 8 (9.20%) patients did not have any neurological deficits after 24 hours. The mean LOS was 120.59 ± 55.29 days for tetraplegia and 115 ± 44.89 days for paraplegia (range 8–273 days).

Causes, annual incidence, and mortality following TSCI The majority of TSCI cases (59 patients (66.6%)) were caused by falls, and 41 (47.1%) of these were falls from height (especially trees). Road traffic accidents lead to 25 (28.7%) cases, while 4 (4.6%) cases were from other forms of trauma including assaults, being fallen on by a heavy object, and being attacked by an animal.

The Kilimanjaro region was estimated to have a population of about 1,910,555 in 2017 based on the Tanzanian national census of 2012 and an annual population growth rate of 2.9%. However, for the year 2017 there was no one aged 10 years or less who were admitted with TSCI. For this reason, those who were in this age group (464,600) were considered as having a negligible risk, and thus the total at-risk population was 1,445,955 persons. The incidence of TSCI for the Kilimanjaro region was 56 cases/1,445,955 persons, giving a rate of at least 38 cases per million population per year. For this period of one year, 15 patients died while in the hospital giving an annual mortality rate of 17.2%.

### Complications

**Figure 1:**
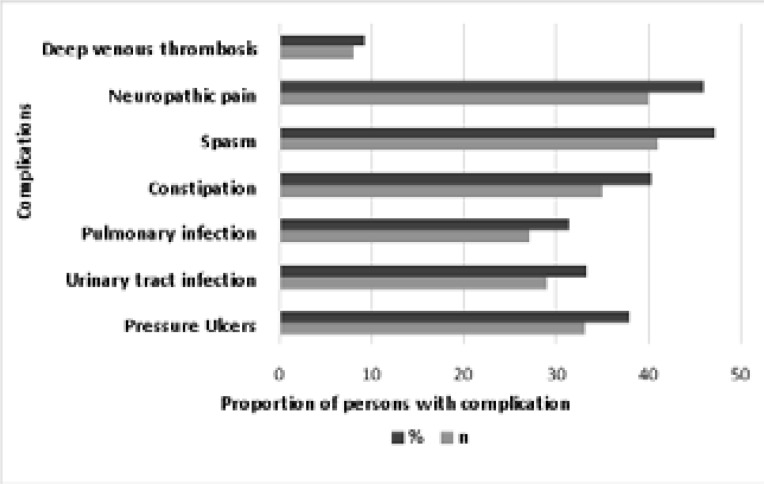
Prevalence of clinical complications

SCI-related complications were identified by clinical observation (PUs), patient reports (spasms and neuropathic pain), and laboratory or radiological confirmations (respiratory infection, UTI, and deep venous thrombosis). Spasms and neuropathic pain were the most prevalent complications, while deep vein thrombosis was the least reported complication as shown in Figure 1.

### Location and time to occurrence and healing of PUs

**Figure 2:**
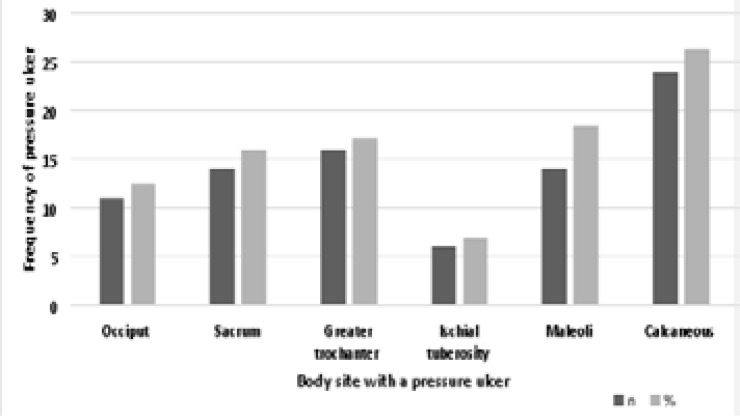
Prevalence of PUs according to body location

PU were followed up to assess their occurrence and healing time. The prevalence of PUs was 37.9%, and the calcaneus was the most common site of occurrence (Figure 2).

As seen in Table 1, PUs was more prevalent for tetraplegia compared to paraplegia, and the longest healing time was observed for PUs on the sacrum and the greater trochanters. Some of the PUs did not heal during hospitalisation (the follow up period).

**Table 1:** Location and time to appearance and healing of PUs in tetraplegia and paraplegia

Location of PU	Number of PUs	Healed PUs on discharge	Unhealed PUs on discharge	Time (days) from admission to appearance of PUs Mean (SD)	Time (days) from appearance to healing of PUs Mean (SD)
**Sacrum**					
Tetraplegia	11	5	6	19.6 (±12.1)	87.6 (±64.3)
Paraplegia	3	3	0	37 (±20.8)	70 (±17.3)
**Greater Trochanter**					
Tetraplegia	13	4	9	29 (±24.1)	71.2 (±65.2)
Paraplegia	3	2	1	29.3 (±9.8)	96
**Ischial Tuberosity**					
Tetraplegia	5	2	3	100.6 (±50.3)	137.5 (±7.8)
Paraplegia	1	1	0	128	156
**Malleolus**					
Tetraplegia	11	8	3	14.5 (±14.7)	25.5 (±12)
Paraplegia	3	2	1	16.3 (±23.2)	45 (±29.7)
**Calcaneus**					
Tetraplegia	17	14	3	22.2 (±13.5)	35 (14.3)
Paraplegia	7	7	0	22.1 (±25)	42.4 (±25)
**Occiput**					
Tetraplegia	11	8	3	14.3 (±10.9)	31.7 (±11.6)
Paraplegia	0	-	0	-	-
**Knee**					
Tetraplegia	7	6	1	34.6 (±28.1)	49.5 (±32/9)
Paraplegia	1	1	0	88	102

## Discussion

This study reports a relatively high incidence of TSCI compared to the results of a systematic review of TSCI in low-income countries 24. The majority of these injuries are due to falls from height and road traffic accidents. Neuropathic pain, spasms, PUs, and UTIs are the most reported complications among persons with TSCI in this region during hospitalisation.

This study registered a higher incidence rate of traumatic spinal cord injury in the Kilimanjaro region than those reported for other low- and middle-income settings^4^,^5^, ^25^, ^26^, but lower than a study in Cape Town, South Africa 3. The majority of these injuries are as a result of falls and road traffic accident. Identification of factors associated with falls and road traffic accidents such as alcohol consumption prior to the incident, environmental and occupational hazards is a pre-requisite to prevention of TSCI 27. Simultaneous with trauma-prevention strategies, there ought to be a development of community-based evacuation and transportation service for injured persons as well as up-to-date trauma registries in these settings in order to obtain more conclusive statistics with fewer missing cases 1.

We report neuropathic pain and spasms as the most prevalent complications in persons with TSCI. These complications lead to massive functional limitations, hinder active rehabilitation, and cause psychosocial problems and lower one's perceived QoL 18. As was shown in studies in New Zealand and Latvia, neuropathic pain normally persists even after discharge from the hospital, suggesting that these patients might have life-long consequences 28, 29. The description and treatment of neuropathic pain is complex, especially when occurring concurrently with spasms 9. It is strongly recommended that pain should be managed appropriately as early as possible to minimize the risk of chronicity 30. However, unavailability and unaffordability of appropriate medications for neuropathic pain and spasms in most low-income settings deters rehabilitation efforts. In addition to the paralysis, these two complications affect movement and functional posture, hence the individual becomes dependent in most activities of daily living. In the long term, chronic pain continues to limit the function of the individual even after discharge from the hospital. At the same time, spasms may keep a limb in a dominant position leading to contractures. For this reason, the QoL of persons with TSCI is continuously affected by pain and spasms even after discharge from the hospital 31, and an interdisciplinary approach is required that includes medication, pain counselling, occupational therapy, and physiotherapy.

PUs is among the most prevalent and deadliest complications globally and accounts to prolonged length of hospital stay and rehabilitation cost 32. Although PUs are still prevalent globally with the same physical risk factors, they are more prevalent in the low-income countries due to various reasons^33^. One of the reasons is insufficient knowledge and skills on early identification and prevention of their occurrence. Second, there is insufficient pressure management equipment such as appropriate mattresses and cushions There are also insufficient numbers of nursing staff to carry out routine turning of patients as recommended. As a result, PUs remains prevalent and contribute significantly to high rehabilitation costs, prolonged hospitalisation, and increased mortality in low and middle-income countries^33^. For a person having PUs, active rehabilitation such as exercising and training for mobility and activities of daily living are limited. For example, PUs around the sacrum and pelvis lead to restricted sitting time, hence the individual spends most of their time lying in bed. Prolonged bed rest not only affects functioning, but also adds to the risk of more PUs elsewhere on the body. Prevention of PUs and successful treatment of those that occur enhances early active rehabilitation and reduces LOS and mortality. Although prevention of PU is costly, the cost of living with or treatinit once it has occurred can be unbearable for persons living in a low-income setting.

Another complication of major concern in this study was UTIs, which are common not only in the low, but also middle and high-income countries^34^. It is almost impossible to avoid UTIs considering the fact that the majority of persons with SCI use catheters for bladder emptying at some point in or throughout their life. However, the method of catheter use and the level of sterility determine the chances of contracting a UTI. For example, a person who uses disposable catheters for intermittent catheterisation would have lower odds of a UTI as compared to a person re-using the same catheter due to dangers of contamination^35^,^36^. In the same way, a person with TSCI who uses an indwelling cathetehas a higher risk of UTI than one who uses clean intermittent catheterisation^37^. Indwelling catheterisation is also known for keeping residual urine in the bladder, which contributes to the formation of bladder stones and to reflux that might damage the kidneys. Properly conducted self-performed intermittent catheterisation or suprapubic catheters for persons who cannot void spontaneously has lower odds for UTIs and hardly any residual urine. Unfortunately, larger populations of persons with TSCI in low-income countries are either using indwelling catheterisation or have to clean and re-use a catheter, which exposes them to a higher risk of UTI. While clean intermittent self-catheterisation is still the most recommended, disposable catheters are too costly in low-income countries and thus efforts should be focused on ensuring cleanliness when using catheters repeatedly. Like other complications, UTIs are also associated with high rehabilitation costs and poor QoL among persons with TSCI globally 38. With symptoms of UTI such as fever, there is limited ability for the patient to engage in exercises or other forms of active rehabilitation.

## Limitation of the study

There are common shortfalls in most epidemiological studies from low-income countries. Like ours, they are normally hospital based and fail to account for patients who die before reaching the hospital. There is also the possibility that some injured persons stay at home and self-medicate or seek alternative traditional treatments. All of these reasons suggest that there is an under-estimation of the magnitude of TSCI in the low-income countries and thus the results of this study are not necessarily exceptional.

## Conclusion

TSCI has relatively high incidence in the north-east region of Tanzania as compared to published studies from other low-income settings. In-hospital complications are substantially prevalent and might reflect the observed long LOS and increased mortality. Such complications adversely affect rehabilitation and activities of daily living leading to dependency and low QoL.
